# Ocular dimensions of the Chinese adolescents with keratoconus

**DOI:** 10.1186/s12886-018-0713-6

**Published:** 2018-02-13

**Authors:** Weijun Jian, Yang Shen, Yingjun Chen, Mi Tian, Xingtao Zhou

**Affiliations:** 1 The Eye and ENT Hospital of Fudan University, Shanghai, China; 2 The Key Lab of Myopia, Ministry of Health, People’s Republic of China, 19 Baoqing Road, Shanghai, Xuhui District China

**Keywords:** Child health (paediatrics), Cornea, Eye (globe), Optics and refraction, Anterior chamber

## Abstract

**Background:**

Adolescent KC is a special segment of the general KC population because an adolescents’s eyes are still susceptible to blurred vision and optical defocus during the sensitive period of ocular and visual development. In the present study, we evaluated the ocular dimensions of 53 KC adolescents.

**Methods:**

One hundred and six KC eyes of 53 (42 boys and 11 girls) KC adolescents (age 15.5 ± 1.7 years, range 11 to 18) were involved in this retrospective study. The eye with more severe KC (Severe Group) of each patient was compared with their less affected eye (Mild Group). Optical axial length (OAL) was measured by optical coherence biometry (IOL-master). Central corneal thickness, anterior chamber depth (ACD), flat keratometry value, steep keratometry value, and maximum keratometry value were assessed with an anterior segment analyzer (Pentacam HR). Anterior segment length (ASL) was manually measured from the 25 scheimpflug images captured by the Pentacam HR with the mean value recorded. The posterior segment length (PSL) was calculated with the formula “PSL = OAL-ASL”.

**Results:**

The mean ACD, OAL, ASL, and PSL values of the Severe Group were 3.51 ± 0.32 mm, 24.76 ± 1.24 mm, 4.01 ± 0.30 mm and 20.76 ± 1.15 mm.While those of the Mild Group were 3.36 ± 0.29 mm, 24.97 ± 1.40 mm, 3.94 ± 0.35 mm and 21.03 ± 1.31 mm. The Severe Group has significantly higher ACD (t = 4.539, *P* < 0.001) value but lower OAL (t = − 3.120, *P* = 0.003) and PSL (t = − 4.537, P < 0.001) values when compared with those of the Mild Group. For the Severe Group, the Kmax values were significantly correlated with the SE values (R = − 0.385, *P* = 0.004), the ACD values (R = 0.375, *P* = 0.006), the ASL values (R = 0.308, *P* = 0.025) and the PSL values (R = − 0.317, *P* = 0.021), but not with the OAL values (R = − 0.220, *P* = 0.114). In the Mild Group, the Kmax values were negatively correlated with the SE (R = − 0.577, *P* < 0.001), OAL(R = − 0.533, P < 0.001), and PSL (R = − 0.523, *P* < 0.001) values, but not with ACD (R = − 0.110, *P* = 0.434) or ASL (R = − 0.182, *P* = 0.192) values.

**Conclusions:**

For adolescent KC, the more keratoconic eyes may be characterized by deeper ACD but shorter OAL and PSL, when compared with the less affected ones.

## Background

Keratoconus (KC) is a chronic and degenerative corneal ectatic disease with an onset during early adulthood or even adolescence [1]. Generally, KC successively affects both eyes. With progressively thinning corneal thickness and steeping corneal curvature, KC would lead to refractive error (refractive myopia and irregular astigmatism) and impaired vision [2]. Nowadays, the improved techniques of corneal topography and in-vivo corneal biomechanical analysis [3–8]significantly increase the ability to diagnose keratoconus. Therefore, it can be easier to detect KC cases in subclinical stages or in the adolescent population [9].

Adolescent KC is a special segment of the general KC population because an adolescents’s eyes are still susceptible to blurred vision and optical defocus during the sensitive period of ocular and visual development [10–12]. However, recent KC studies have been mostly focused on investigating the structural changes in the anterior segment, including corneal shape (corneal curvature, antertior corneal elevation, posterior corneal elevation, corneal thickness), anterior chamber depth, anterior chamber angle [13–22]. The effect of KC on the ocular axial length in the adolescent population is poorly understood. In the present study, we evaluated the ocular dimensions of 53 KC adolescents.

## Methods

### Participants

One hundred and six KC eyes of 53 KC adolescents (42 boys and 11 girls) whose refractive error have been or have never been corrected by spectacles were recruited in this retrospective study from August 2015 to September 2016 at the Department of Ophthalmology of Eye and ENT hospital, Fudan University. The mean age was 15.5 ± 1.7 years (range 11 to 18). The eye with higher Kmax value (Severe Group) of each patient was compared with their lower Kmax value eye (Mild Group).

### Ophthalmologic examinations

**Fig. 1:**
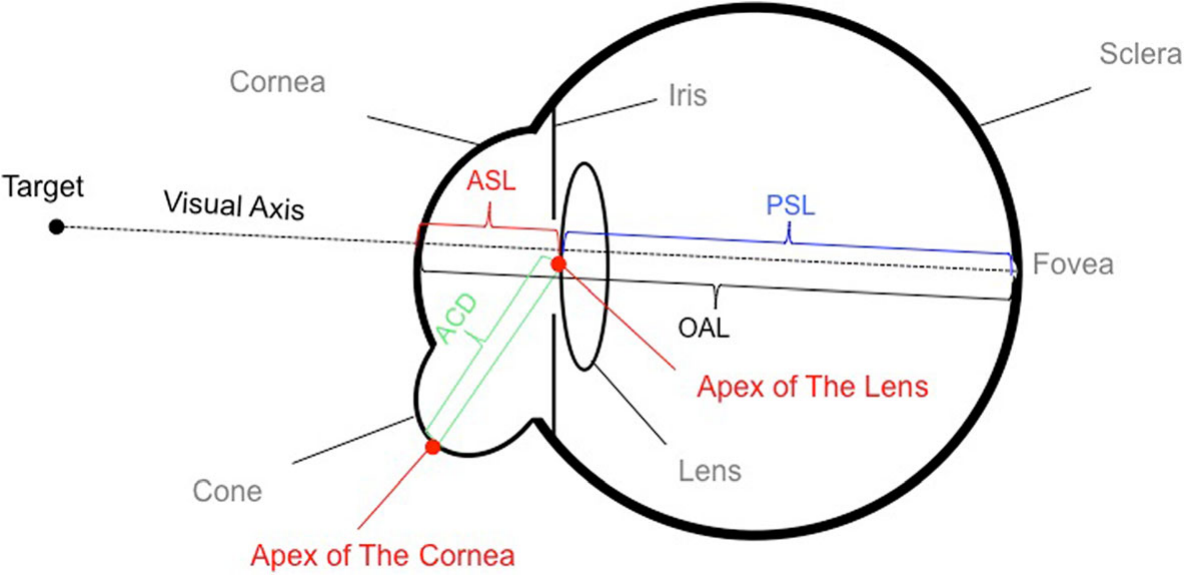
The posterior segment length (PSL), which refers to the distance from the anterior capsule of the lens to the retinal pigment epithelium layer of the macula, was calculated with the formula “PSL = OAL-ASL”

Each participant underwent manifest refractions (cycloplegic and non-cycloplegic) and best spectacles corrected distance acuity (BSCDVA) examinations. The anterior segmental parameters including flat keratometry value (K1), steep keratometry value (K2), maximum keratometry value (Kmax), central corneal thickness (CCT) and anterior chamber depth (ACD) were assessed with an anterior segment analyzer (Pentacam HR, Typ70900, Oculus Optikgeräte GmbH, Wetzlar, Germany) at a sitting position. Images with an ‘OK’ in quality specification (QS) were analyzed. An optical coherence biometry (IOL-master 1322–734, Carl Zeiss Meditec AG, Jena, Germany) was applied to evaluate the optical axial length (OAL) value along the visual axis (line connecting the fixation point to the fovea, specifically from the anterior surface of the cornea to the retinal pigment epithelium layer of the fovea). Five measurements were continuously obtained from each eye at a sitting position, with the mean value of the five measurements calculated automatically. The anterior segment length (ASL) of each eye (from the anterior surface of the cornea to the anterior capsule of the lens along the optical axis) was manually measured from the 25 scheimpflug images of each eye captured by the Pentacam HR, with the mean value recorded. The posterior segment length (PSL), which refers to the distance from the anterior capsule of the lens to the retinal pigment epithelium layer of the macula, was calculated with the formula “PSL = OAL-ASL” (Fig. 1).

### Data analysis and statistical evaluation

Statistical analysis was performed using SPSS 19 (SPSS Inc., IBM). Normality check was conducted using the Kolmogorov–Smirnov Z test. All these parameters of the eyes in the Severe Group (with higher Kmax value) were compared with those of the eyes in the Mild Group. Paired-t test was used for the normal distribution data. If the data distribution was not normal, Wilcoxon signed-rank test was employed for the analysis. The potential correlations between SE, Kmax and OAL were evaluated with Pearson correlation tests. Cut-off *P* values were 0.05.

## Results

### Refraction and the main topographic parameters

The mean manifest refraction (cycloplegic) spherical equivalent (SE) of the eyes in the Severe Group was − 8.26 ± 4.72 diopters (D), which was much more myopic (*P* < 0.05) than those in the Mild Group. (− 5.62 ± 3.52D). As listed in Table 1, the mean CCT value of the Severe Group was 459.3 ± 45.8 μm, significantly lower (t = − 7.933, *P* < 0.001) than that of the Mild Group. (504.3 ± 39.9 μm) The mean values of K1, K2 and Kmax values of the Severe Group were 50.72 ± 7.60D, 55.56 ± 8.15D and 63.79 ± 11.49D, respectively. They were significantly higher than those of the Mild Group (t = 6.922, *P* < 0.001; t = 8.614, *P* < 0.001; t = 9.791, *P* < 0.001, respectively).

**Table 1:** Main Ocular Parameters (*n* = 106)

Variables	Worse Eyes (*n* = 53)		Fellow Eyes (n = 53)		*t* value^a^	*P* value	*D*-value Mean ± SD
	Mean ± SD	Range	Mean ± SD	Range			
K1 (D)	50.72 ± 7.60	41.40 to 70.70	43.77 ± 2.95	35.50 to 54.30	6.922	< 0.001^b^	6.95 ± 7.30
K2 (D)	55.56 ± 8.15	43.20 to 74.60	46.38 ± 3.62	41.30 to 56.10	8.614	< 0.001^b^	9.18 ± 7.75
Kmax (D)	63.79 ± 11.49	45.20 to 86.90	50.03 ± 6.57	42.10 to 69.00	9.791	< 0.001^b^	13.76 ± 10.23
CCT (μm)	477.5 ± 42.7	377 to 576	515.0 ± 37.5	424 to 587	−8.350	< 0.001^b^	−37.57 ± 32.75
TCT (μm)	459.3 ± 45.8	331 to 541	504.3 ± 39.9	404 to 583	−7.933	< 0.001^b^	−45 ± 41.30
ACD (mm)	3.51 ± 0.32	2.62 to 4.39	3.36 ± 0.29	2.65 to 3.91	4.539	< 0.001^b^	0.15 ± 0.24
OAL (mm)	24.76 ± 1.24	22.26 to 27.28	24.97 ± 1.40	22.37 to 27.70	−3.120	0.003^b^	−0.21 ± 0.49
ASL (mm)	4.01 ± 0.30	3.19 to 4.88	3.94 ± 0.35	3.19 to 5.46	1.876	0.066	0.07 ± 0.25
PSL (mm)	20.76 ± 1.15	18.58 to 23.26	21.03 ± 1.31	18.50 to 23.54	−4.537	< 0.001^b^	−0.27 ± 0.44

*MRSE* = manifest refraction spherical equivalent; *D* = diopter; *K1* = flat keratometry value;*K2* = steep keratometry value; *Kmax* = maximum keratometry value; *CCT* = central corneal thickness; *TCT* = thinnest corneal thickness, *ACD* = anterior chamber depth; *OAL* = optical axial length; *ASL* = anterior segment length; *PSL* = posterior segment length

^a^Paird t-test

^b^Significant difference was detected between groups

### Main ocular dimensional parameters

**Fig. 2:**
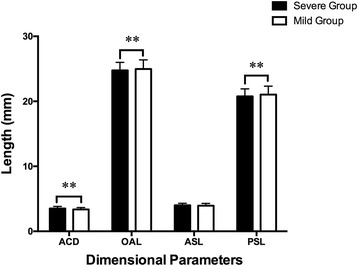
No statistically significant difference was detected (t = 1.876, *P* = 0.066) in the mean ASL values between groups

The mean ACD, OAL, ASL, and PSL values of the Severe Group were 3.51 ± 0.32 mm, 24.76 ± 1.24 mm, 4.01 ± 0.30 mm and 20.76 ± 1.15 mm, respectively. While those of the Mild Group were 3.36 ± 0.29 mm, 24.97 ± 1.40 mm, 3.94 ± 0.35 mm and 21.03 ± 1.31 mm, respectively. The mean ACD value of the Severe Group was significantly higher than the Mild Group (t = 4.539, *P* < 0.001), but the mean OAL and PSL values of the Severe Group were remarkably lower, when compared with the fellow eyes (t = − 3.120, *P* = 0.003; t = − 4.537, *P* < 0.001, respectively). No statistically significant differences were detected (t = 1.876, *P* = 0.066) in the mean ASL values between groups (Fig. 2).

### Correlations between the ocular dimensional parameters

**Fig. 3:**
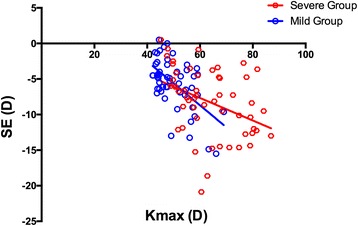
The Kmax values significantly correlated with the SE values

**Fig. 4:**
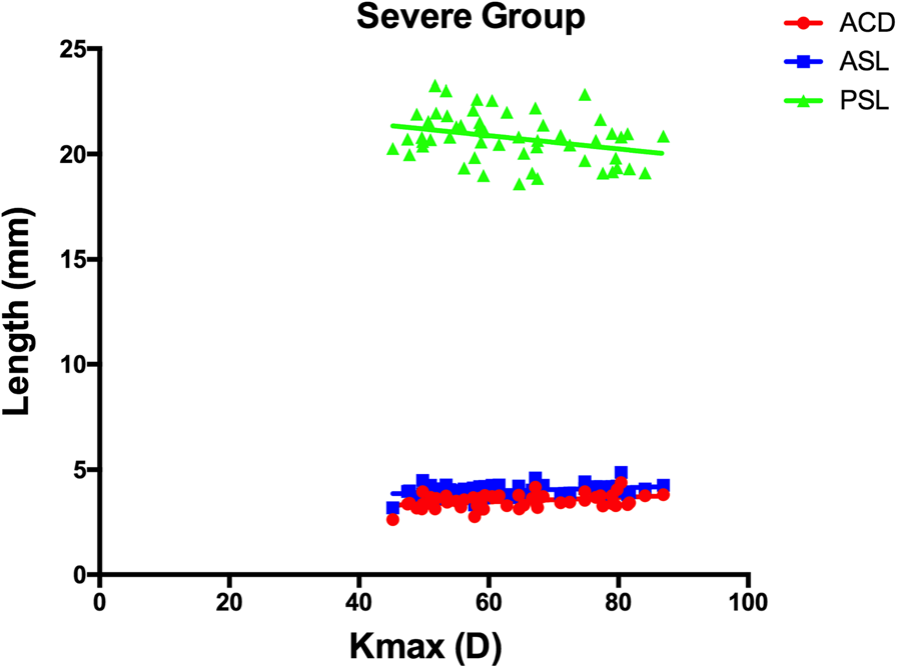
In Severe Group, the Kmax values significantly correlated with the ASL values (*R* = 0.308, *P* = 0.025) and the PSL values. (*R* = − 0.317, *P* = 0.021)

**Fig. 5:**
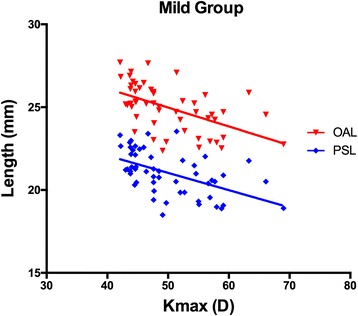
In Mild Group, The Kmax values negatively correlated with OAL(*R* = − 0.533, *P* < 0.001), and PSL (*R* = − 0.523, *P* < 0.001) values

For the Severe Group, as shown in the Figs. 3 and 4, the Kmax values were significantly correlated with the SE values (R = − 0.385, *P* = 0.004), the ACD values (R = 0.375, *P* = 0.006), the ASL values (R = 0.308, *P* = 0.025) and the PSL values (R = − 0.317, *P* = 0.021), but not with the OAL values (R = − 0.220, *P* = 0.114). In the Mild Group, as demonstrated in the Figs. 3 and 5, the Kmax values were negatively correlated with the SE (R = − 0.577, *P* < 0.001), OAL(R = − 0.533, P < 0.001), and PSL (R = − 0.523, *P* < 0.001) values, but not with ACD (R = − 0.110, *P* = 0.434) or ASL (R = − 0.182, *P* = 0.192) values.

## Discussion

Keratoconus commonly leads to refractive error (myopia and irregular astigmatism) and visual impairment, which is very hard to fully correct using spectacles. Evidence has shown that, due to the ectasia of the cornea, adult keratoconic eyes are characterized by longer axial length and deeper anterior chamber depth, compared with normal eyes [23]. But our knowledge on the ocular dimensions of adolescent KC patients is still limited.

In the present study, we investigated corneal topographies and ocular dimensions of 53 adolescent KC patients. Similar to adult KC, the more severely affected eyes of these adolescent KC patients have steeper corneal curvature, thinner corneal thickness and higher myopia when compared with their less affected ones. Moreover, the ACD values of the more severely affected eyes are higher than their counterparts, while the difference in ASL values between the severe and fellow eyes is not significant. A possible explanation is that the Pentacam HR evaluates ASL along the visual axis, but measures ACD from the corneal apex (the steepest point of the anterior corneal surface). It is known that, for a young keratoconic eye, the steepest point of the cone (the corneal apex) is most frequently located in the paracentral or peripheral area of the cornea [24]. In contrast, the central cornea, especially the visual axial area may be less affected compared with the corneal apex.

It is noticeable that the PSL and OAL values of the Severe Group were shorter than those of the Mild Group. Further analysis showed that, for the eyes in the Severe Group, the values of Kmax were positively correlated with the values of ACD (consists with the findings of Mas-Aixala E [25] and Safarzadeh M [26]) but negatively correlated with the values of PSL. This indicates that, in these severe cases, KC may affect both the dimensions of the anterior (positive correlation) and posterior (negative correlation) segments. We also noticed that Kmax did not correlate with OAL in the Severe Group. One possible reason is that the shortened PSL partially compensated the deepened ACD, which affected the overall changes of the OAL.

In the Mild Group, both the PSL and OAL values were negatively correlated with the Kmax value. This implies that for the mild cases, KC mostly affects the posterior, rather than the anterior segment of an eye.

It is interesting that the mean OAL and PSL values of the Severe Group were much lower than those of the Mild Group. We hypothesize that severe cases of KC may lead to more severe myopia and astigmatism, which may induce stronger myopic defocus signals when compared with less affected eyes. It has been reported that peripheral myopic defocus could thicken choroid and inhibit eye growth in birds [27], primates [28] and even in human beings [29] during their emmetropization. As the cone of a keratoconic cornea is mostly located in the peripheral area, the peripheral retina may acquire the myopic defocus signal, and then trigger the mechanism, thus leading to the shortening of the PSL and OAL among those adolescent KC patients.

In our study, it was found that KC not only affected the refractive status but also the development of the axial length in adolescents. Because the development of the eyeball is not still in adolescents, the axial length is constantly changing. It refers that KC may lead more complex influence in adolescents. Therefore, when we dealing with adolescent KC patients, the axial length is also an important examination besides refractive status and corneal topography.

One limitation of our study is that the IOL-master was unable to evaluate the choroid thickness. Further studies are essential to determine the effects of optical defocus caused by kerotoconus on choroid thickness. Another limitation is that although most of the KC adolescents in the present study were initially diagnosed and never underwent any optical corrections, some of them had already been treated by spectacles. As keratoconus progresses silently and occurs successively with both eyes, the exposure time and dose of optical defocus in the earlier-onset eye (the more severely affected eye) are unquestionably longer and greater than the later-onset one.

## Conclusions

In conclusion, for KC adolescents, the more affected keratoconic eyes may be characterized by deeper ACD but shorter OAL and PSL, when compared with the less affected ones.

## Abbreviations

ACD: Anterior chamber depth
ASL: Anterior segment length
BSCDVA: Best spectacles corrected distance visual acuity
CCT: Central corneal thickness
KC: Keratoconus
Kmax: Maximum keratometry value
OAL: Optical axial length
PSL: Posterior segment length
